# Quality Report: Postoperative Guideline Implementation Reduces Length of Stay after Fontan Procedure

**DOI:** 10.1097/pq9.0000000000000661

**Published:** 2023-06-07

**Authors:** Virginia Cox, Stephen Hart, Diane Hersey, Jennifer Gauntt, Sergio Carrillo, Patrick McConnell, Janet Simsic

**Affiliations:** From The Heart Center at Nationwide Children’s Hospital, Columbus, Ohio.

## Abstract

**Introduction:**

Patients following the Fontan procedure have a physiology that results in prolonged pleural effusion, often delaying hospital discharge. The hospital length of stay (LOS) of patients following the Fontan procedure at our institution was significantly longer than the Society of Thoracic Surgery benchmark. This quality improvement project aimed to decrease hospital LOS in patients following the Fontan procedure from a baseline of 23 days to 7 days by January 1, 2021, and sustain indefinitely.

**Methods:**

We implemented standardized postoperative clinical practice guidelines in April 2020. We designed guidelines using previously published protocols. Key features included an ambulatory PleurX drain (BD, Franklin Lakes, N.J.), diuresis with fluid restriction, and pulmonary vasodilation with supplemental oxygen and sildenafil. All patients were discharged from the hospital with a PleurX drain in place. We compared clinical outcome variables before and after guideline implementation. As a balancing measure, we tracked 30-day readmissions.

**Results:**

One hundred seven patients underwent the Fontan procedure before guideline implementation from January 2015 to January 2020, with an average hospital LOS of 23 days. Postguideline implementation, 35 patients underwent the Fontan procedure from April 2020 to July 2022, with an average hospital LOS of 8 days in 2020, which further improved to an average hospital LOS of 7 days. There was no change in 30-day readmission after guideline implementation (24% pre versus 23% post; *P* = 0.86).

**Conclusion:**

Implementing clinical practice guidelines for patients following the Fontan procedure led to an over 50% reduction in hospital LOS without increasing 30-day readmission.

## INTRODUCTION

The Fontan procedure, usually performed in the toddler age group, is the final stage of single-ventricle palliation. Following the Fontan procedure, patients have unique single-ventricle physiology with passive, nonpulsatile extracardiac pulmonary blood flow in one circuit and a single pumping chamber providing systemic blood flow in the other. This physiology often results in prolonged postoperative pleural drainage, which can delay hospital discharge. Numerous studies have described potential etiologies for pleural effusion after the Fontan procedure.^1–3^ Several mechanisms have been the target of postoperative protocols to reduce the duration of pleural drainage, including elevated pulmonary artery pressures, reduced ventricular function, mechanical ventilation, atrioventricular valve regurgitation, surgical technique, and fluid overload.^4–6^

After reviewing national benchmarks, hospital length of stay (LOS) at Nationwide Children’s Hospital (NCH) following the Fontan procedure was significantly longer than the Society of Thoracic Surgery benchmark (NCH average 23 days versus Society of Thoracic Surgery average of 14.4 days).^7^

This finding was the impetus for this quality improvement initiative. In addition, after reviewing our patient population, we found that most patients recovered from Fontan surgery after about 5–7 days and only remained in the hospital secondary to their continuous chest tube drainage. Therefore, we ambitiously sought to reduce our LOS to 7 days.

Clinical practice guidelines are tools to reduce practice variation, improve the quality of care, and contain costs.^8^ According to the Institute of Healthcare Improvement’s strategy for designing systems, the first step is to create a simple, standardized approach or guideline that is minimally controversial.^9,10^ With this in mind, we reviewed the literature for clinical practice guidelines following the Fontan procedure,^5,6^ to create our institutional guidelines for this patient population. Key features included ambulatory PleurX drain (BD, Franklin Lakes, N.J.), inpatient aggressive diuresis with fluid restriction, and pulmonary vasodilation with supplemental oxygen and sildenafil. This quality improvement project aimed to develop and implement standardized inpatient postoperative clinical practice guidelines for patients following the Fontan procedure, specifically aiming to decrease hospital LOS from a baseline of 23 days to 7 days by January 1, 2021, and sustain indefinitely.

## METHODS

### Ethical Considerations

This quality improvement work involved the development of standardized inpatient postoperative Fontan clinical practice guidelines. No interventions involved comparing multiple devices or therapies, and patients were not subjected to randomization. Quality improvement team members reviewed patient medical records as per their normal responsibilities. We did not evaluate race and ethnicity; therefore, data regarding race and ethnicity are not included in this quality initiative. Per our institutional policy, this project does not meet the definition of human subject research. Therefore, institutional review board approval was not required.

### Setting

NCH is an academic, nonprofit, freestanding children’s hospital in Columbus, Ohio. The Heart Center at NCH comprises a 20-bed cardiothoracic intensive care unit with over 600 admissions per year and a 24-bed cardiac acute care unit with over 1300 admissions annually. These units are staffed by a multidisciplinary team of critical care and cardiology physicians in the cardiothoracic intensive care unit (n = 11), cardiology physicians in the acute care unit (n = 9), pediatric cardiothoracic surgeons (n = 4), advanced practice nurse practitioners (n = 21), dedicated clinical pharmacists (n = 2), registered nurses (cardiothoracic intensive care unit n = 61; acute care unit n = 58), respiratory therapists (n = 21), and rotating resident and fellow trainees. In addition, the Heart Center also employs dedicated clinical dieticians, physical and occupational therapists, child life specialists, social workers, and discharge planners who are integral to patient care.

### Planning the Intervention

**Fig. 1.**
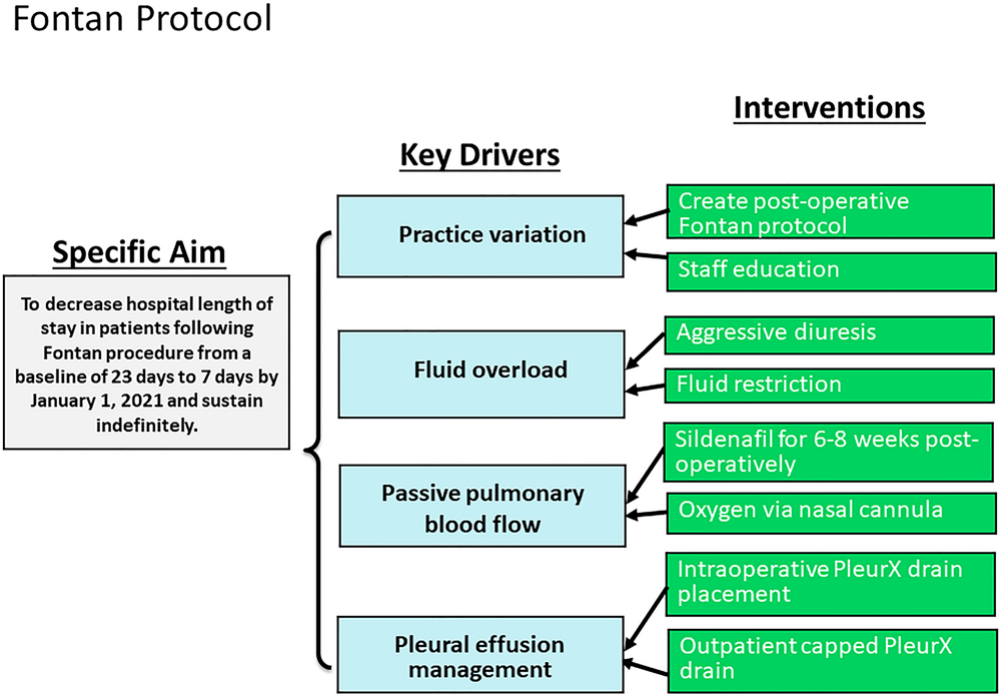
Key driver diagram.

A multidisciplinary team developed and implemented standardized guidelines for postoperative clinical management in the cardiothoracic intensive care unit and acute care unit for patients following the Fontan procedure. Cardiothoracic intensive care unit and acute care unit physicians, inpatient and outpatient advanced practice nurse practitioners, a cardiothoracic surgeon, and representatives from the Center for Clinical Excellence (quality and safety department) were the primary team members. A SMART (specific, measurable, achievable, relevant, and time-bound) aim statement and key driver diagram were created (Fig. 1). Practice variation, fluid management, passive pulmonary blood flow, and pleural effusion management are key drivers.

### Interventions

**Fig. 2.**
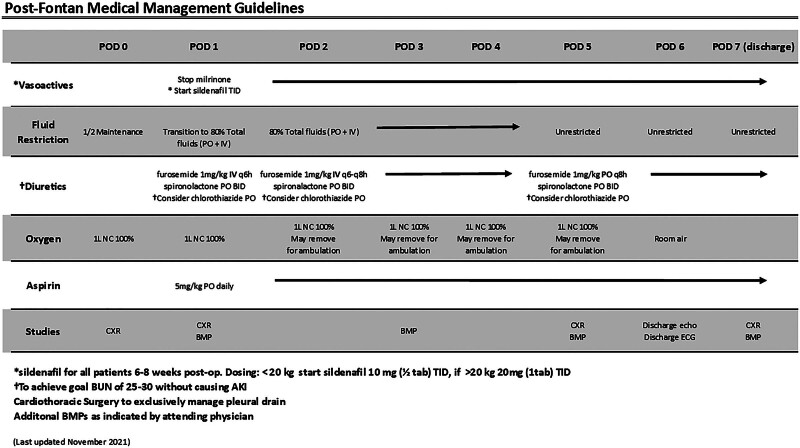
Fontan guideline. BID, twice per day; BMP, basal metabolic panel; ECG, electrocardiogram; echo, echocardiogram; IV, intravenous; NC, nasal cannula; PO, by mouth; q6h, every 6 hours; q8h, every 8 hours; TID, 3 times per day.

Standardized postoperative clinical practice guidelines (Fig. 2) are developed and implemented to reduce variability and standardize the in-hospital postoperative management of patients following the Fontan procedure. At our institution, the routine is to perform a nonfenestrated Fontan procedure. We crafted the guideline to mitigate the risk factors for prolonged pleural drainage as we felt that prolonged pleural drainage was driving our LOS. These guidelines uniquely include placement of the PleurX catheter, in addition to assuring tight control of postoperative fluid balance with fluid restriction and aggressive diuretics and lowering pulmonary vascular resistance to encourage passive forward pulmonary blood flow with the use of sildenafil and oxygen. The diuretics are adjusted to achieve a blood urea nitrogen (BUN) level of 25–30 mg/dL. Using BUN for diuretic management is based on concerns from the group that utilizing fluid balance alone might result in acute kidney injury secondary to aggressive diuretics and likely inaccurate recording of the fluid balance (the ins and outs) in this toddler population. Therefore, following BUN might reduce potential harm from aggressive diuretic management.

Both pre- and postguideline patients following the Fontan procedure are started on milrinone intraoperatively to prevent postoperative low cardiac output syndrome. Milrinone was typically discontinued on postoperative day (POD) 1. All providers did not consistently utilize preguidelines, sildenafil, and oxygen. Diuretic management and fluid restriction were at the discretion of the physicians, thus leading to variation in practice. Preguidelines, the pleural drainage strategy was a traditional chest tube, and the patient remained in the hospital until this chest tube was removed. Postguidelines, a PleurX drain is placed intraoperatively in the right pleural space at the time of the Fontan procedure with the intent for the patient to go home with this drain in place. The PleurX drain is capped for 48 hours once drainage is less than 350 ml (usually on POD 5). All patients are discharged with the drain in place when they can tolerate the pleural drain capped for 48 hours before discharge (goal POD 7). Tolerating PleurX drain capping is defined as no increase in work of breathing or decrease in systemic saturations after 48 hours of capping. The patients returned to the outpatient clinic 48 hours after hospital discharge for a chest radiograph (CXR) and evaluation by the cardiothoracic advanced nurse practitioner. At this visit, the PleurX catheter is also drained. Based on pleural drainage and clinical assessment, patients are followed in the outpatient clinic every 48–72 hours until pleural drainage decreases, and the patient returns to baseline activity, medication, and enteral intake. At that time, there is a discussion between the cardiothoracic advanced nurse practitioner, cardiologist, and cardiothoracic surgeon regarding PleurX removal. Diuretic management in the outpatient setting is at the discretion of the outpatient cardiologist.

Before the rollout of the guidelines, we provided in-person education to unit staff during regularly scheduled unit staff meetings regarding the guideline and goals of the project. Periodic updates are provided and felt to be the key to eliciting and continuing buy-in from the group. A member of the quality improvement team also sends an email to unit staff the day before each patient’s scheduled operation to remind staff of the guideline. In addition, the guideline is printed and attached to the patient’s bed before leaving the operating room as a visual reminder about guideline components.

We instituted the above interventions for all patients undergoing the Fontan procedure beginning in April 2020 (no patients underwent the Fontan procedure between January and April 2020). We reviewed compliance and clinical outcomes data following the first 5 patients and the first 15 patients as part of the Plan-Do-Study-Act (PDSA) cycles. The first PDSA cycle was to ensure patient safety with guideline implementation. After carefully reviewing the first 5 patients, the multidisciplinary team felt the guidelines were safe. No patient had a BUN greater than 30. No patient had hypotension from sildenafil administration. Following the second PDSA cycle, we redefined fluid restriction from 80% enteral intake to 80% intravenous plus enteral intake, as some patients remained on intravenous fluids longer than expected, secondary to insufficient enteral intake. Additionally, we changed furosemide from every 6 hours or every 8 hours on POD 1 to furosemide every 6 hours standardly.

### Clinical Team Structure

Three cardiothoracic surgeons were consistent throughout the study; a fourth cardiothoracic surgeon joined the team in 2018. The 9 acute care attending physicians were the same throughout the study. In 2019, these 9 attending physicians started covering the weekends. Before that, the weekends were distributed among a larger group of cardiologists. Seven cardiac intensivists were consistent throughout the study period. Four additional cardiac intensivists joined the team during the study time: 2 in 2018, 1 in 2020, and 1 in 2021.

### Method of Evaluation and Analysis

We included all patients who underwent the Fontan procedure during the study period in the analysis except those who died before hospital discharge (n = 2 preguidelines and n = 0 postguidelines). The primary outcome measure was hospital LOS. We used a statistical process control I-MR chart with standard upper and lower control limits (±3 standard deviations) to follow hospital LOS for each patient starting in January 2015. January 2015 through January 2020 was used as the process baseline. We applied Nelson’s rules for special cause variation to identify a centerline shift in a process stage mean.^8^ The process measure is compliance with each of the elements of the clinical practice guideline. We tracked guideline element compliance via manual retrospective chart review for each intervention group patient. The balancing measure was 30-day readmission to ensure that earlier hospital discharge did not increase rehospitalization. Statistical analysis was performed using χ2 for categorical data and the Mann-Whitney test for nonnormally distributed continuous data.

## RESULTS

**Fig. 3.**
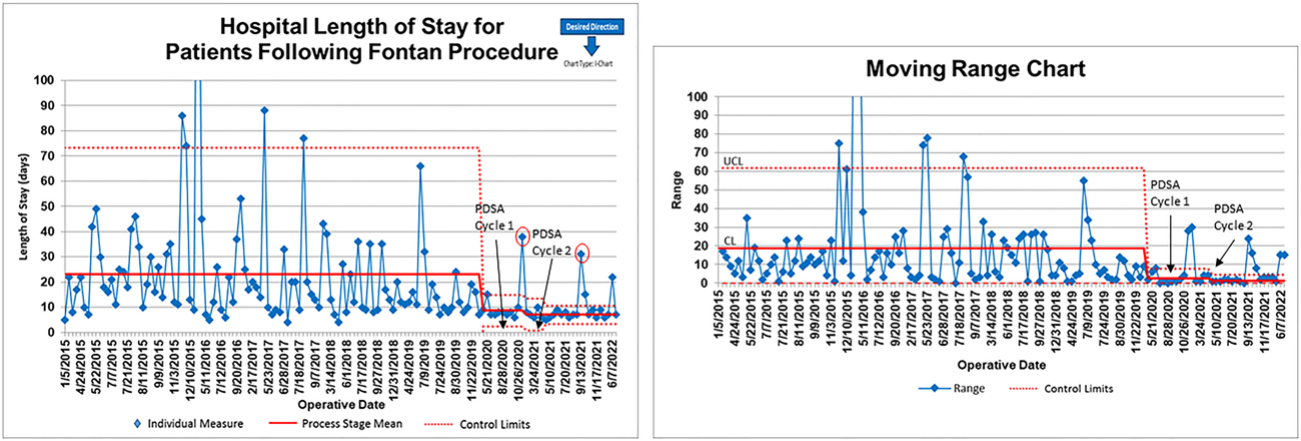
I-MR chart of hospital LOS for patients following the Fontan procedure.

Demographic and outcome variables pre- and postguidelines are shown in Table 1. The primary outcome measure, hospital LOS, is shown in Figure 3. One hundred and seven (107) patients underwent the Fontan procedure preguidelines from January 2015 to January 2020, with an average hospital LOS of 23 ± 27 days (median 16 days; range 4–238 days). Thirty-five patients have undergone a nonfenestrated Fontan procedure postguidelines (since February 2020), with an initial average hospital LOS of 8 days, which further improved to 7 days, an over 50% reduction in hospital LOS.

**Table 1.** Demographics and Outcome Variables Pre- and Postintervention

Variables	Baseline	Intervention	*P*
N=	107	35	
Age (y)	3.8 ± 2.8	3.5 ± 1.4	0.6
Median (range)	3.1 (1.5–22.3)	3.1 (1.4–7.9)	
Weight (kg)	15.8 ± 10.2	15 ± 3.2	0.3
Median (range)	13.9 (9.5–107.2)	14 (10.5–28.6)	
Hospital LOS (d)	23.1 ± 27	9.7 ± 7	**<0.0001**
Median (range)	16 (4–238)	7 (5–38)	
Readmission within 30 days	26 (24%)	8 (23%)	0.86

Bold indicates statistically significant values.

Two patients postguidelines had hospital LOS greater than 30 days, as seen on the I-MR chart in Figure 3. Both patients had chylous pleural drainage. These 2 patients are the impetus for discussing additional management strategies for chylous pleural drainage following the Fontan and have led to an ongoing project with our lymphatic team. However, these 2 patients did not influence the current guideline PDSA cycles.

### Guideline Components

BUN was greater than 30 mg/dL at some point in 12 patients. Those patients had a reduction in diuretics secondary to elevated BUN. No patient suffered from acute kidney injury requiring dialysis. The average duration of indwelling PleurX drain was 41 days (median, 34 days; range, 8–188 days). One patient had accidental PleurX drain dislodgement at home despite appropriate maintenance provided by the patient’s caregiver. There was no harm to the patient, no evidence of pneumothorax on the CXR, and no need for PleurX drain replacement.

### Balancing Measure

Thirty-day readmission is tracked as a balancing measure. There was no difference in readmission pre- and postguidelines. There were 26 hospital readmissions in 107 patients (24%) preguidelines and 8 hospital readmissions in 35 patients (23%) postguidelines, *P* = 0.86. Four of the 8 readmissions had respiratory complaints that did not improve following pleural fluid removal from the PleurX drain. Two patients were readmitted with pleural effusions on the side without the PleurX drain. One patient was readmitted for fever and one for emesis.

### Compliance

Compliance with each component of the standardized postoperative practice guidelines is in Table 2. All patients are started on sildenafil for pulmonary vasodilation on POD 1; 97% are started on intravenous diuretics POD 1. Compliance with the other guideline components is variable. Nasal cannula oxygen and fluid restriction challenged the group and/or the patients. Seven toddlers did not tolerate nasal cannula oxygen, and it was, therefore, discontinued. Eight patients had liberalization of their fluid status sooner than the guideline stated. This finding was likely secondary to the provider and/or parent giving in to the toddler’s desires. Several patients did not start aspirin POD 1 as they had not started enteral nutrition.

**Table 2.** Guideline Component Compliance

Guideline Component	Compliance, N = 35 (%)
Sildenafil	35/35 (100)
Fluid restriction	27/35 (77)
Diuretics	
POD 1 (IV q6h)	34/35 (97)
POD 5 (change to PO q8h)	23/35 (66)
Oxygen	17/35 (49)
ASA	28/35 (80)

IV, intravenous;

PO, by mouth; q6h, every 6 hours; q8h, every 8 hours.

## DISCUSSION

We sought to reduce hospital LOS after discovering that our performance was below the national average by developing and instituting clinical practice guidelines. Practice guidelines are systematically developed management protocols intended to assist with decisions regarding care for certain medical situations.^11^ These guidelines are intended to improve decision-making and optimize patient outcomes.^9,11–14^ We built our guidelines on previously described protocols with several key features, including the use of ambulatory PleurX drains, aggressive diuresis, and fluid restriction, and maximization of pulmonary vasodilation with supplemental oxygen and sildenafil.^4,5^ Our postoperative inpatient clinical practice guidelines for patients following Fontan procedure significantly reduced hospital LOS without impacting 30-day readmissions.

### Indwelling PleurX Drain

After a review of our preguideline data, we felt that our chest tube drainage was the driving force behind our LOS. Therefore, we chose to address this issue creatively. The cardiothoracic surgeons placed PleurX catheters intraoperatively in the right pleural space as part of the initiative. The PleurX catheter is a small-bore tunneled drain designed for intermittent ambulatory drainage of the pleural space through a 1-way valve. The PleurX drain has proven effective for pleural fluid drainage, and complication rates have been low in adults.^15–17^ This study is the first to report the ambulatory use of the PleurX drain in the post-Fontan patient population. In the current study, all patients are discharged with a PleurX drain in the right pleural space. The patients returned to the clinic every couple of days to have their pleural fluid drained. The PleurX drain is removed once the pleural fluid no longer accumulates. We recognize that patients discharged with capped PleurX drains may be the most important intervention for the decreased hospital LOS. Although other published studies on post-Fontan guidelines reported success in decreasing hospital LOS by focusing on diuresis, fluid restriction, and a low-fat diet to decrease the volume and duration of pleural fluid drainage,^4–6^ this initiative took a unique approach to decrease hospital LOS by managing the pleural fluid in the outpatient setting.

### Sildenafil and Oxygen

Low pulmonary vascular resistance is important to the single-ventricle circulation without a pulmonary pump. In a prospective study by Tunks et al, 9 patients with Fontan circulation underwent hemodynamic catheterization.^5,18^ They found reduced pulmonary artery pressures, a trans-pulmonary gradient, and an indexed pulmonary vascular resistance after intravenous sildenafil.^18^ Pulmonary blood flow increased, and ventilation improved.^18^ Improvements in pulmonary hemodynamics translate to improved pulmonary blood flow and, thus, cardiac output. Strategies to improve cardiac output may have clinical utility in the acute postoperative period and over the long term to improve functional status. However, routine postoperative administration of sildenafil after the Fontan procedure was not associated with an improvement in clinical outcome, including postoperative chest tube output or hospital LOS.^19,20^ Secondary to the hemodynamic appeal of sildenafil, despite the lack of positive data in the literature, the multidisciplinary group chose to add sildenafil to the guidelines. The guidelines included supplemental nasal cannula oxygen (1 L/min) when in bed, regardless of systemic saturations, for the pulmonary vasodilatation effect until hospital discharge. This component was challenging as continued use of oxygen was not previously standard practice, and some children did not tolerate the nasal cannula.

### Diuretics and Fluid Restriction

Alterations of intravascular and total body fluid in pediatric patients following cardiac surgery result from intrinsic myocardial dysfunction, neuroendocrine response, renal dysfunction, inflammatory cascade induced by cardiopulmonary bypass, increased capillary permeability, decreased plasma oncotic pressure, increased tissue osmotic pressure, and endothelial dysfunction.^21–24^ Capillary leak and low cardiac output syndrome are more pronounced during the first 6 to 18 hours following cardiac surgery.^24,25^ The resulting fluid overload may contribute to acute kidney injury, prolonged mechanical ventilation, need for vasoactive support, prolonged intensive care unit and hospital LOSs, and mortality.^21,26–28^ To combat these adverse effects, reducing extravascular fluid accumulation with diuretics is one of the management mainstays for pediatric patients following cardiac surgery. Standardizing diuretic management was challenging. We chose an aggressive diuretic strategy (furosemide every 6 hours intravenous with spironolactone and hydrochlorothiazide as needed) and monitoring BUN periodically as a safety measure for over-diuresis. BUN was an objective number that the group could all agree on. Compliance with the strict diuretic management guideline was challenging. Only 66% were on enteral furosemide every 8 hours on POD 5 per the guidelines. Perhaps, the guidelines were too rigid and did not allow enough patient variability.

Regarding fluid and enteral restrictions, several patients required intravenous fluids for a longer timeframe, secondary to inadequate enteral intake. Most patients had less than 80% of total daily fluids combined with intravenous and enteral feeds. Some patients had their fluid restriction liberalized sooner than the guideline called for, likely secondary to the provider and/or parent responding to the toddler’s desire to drink more.

### Limitations and Lessons Learned

We implemented all the components of this project simultaneously with no attempt to ascertain which intervention was most or least effective. There was no baseline data for aspirin, sildenafil, or diuretic use preguideline implementation; therefore, we cannot comment on practice changes for these items. Diuretic management in the outpatient setting remained at the discretion of the outpatient cardiologist. Parental satisfaction with the guidelines was not assessed (a limitation and potential point for future study). Further work is needed to investigate the full impact of at-home PleurX drains, including the benefits of earlier discharge, the potential burdens of increased outpatient follow-up care, and parental preference. This study is limited to a single center with a dedicated Center for Clinical Excellence that allocates resources for quality improvement projects. Institutions without these resources may have difficulty duplicating our work.

Key quality strategies that contributed to the successful process included (1) a multidisciplinary team to question and explore current practice; (2) creating simple guidelines to standardize practice; (3) staff accountability for implementation and outcomes; (4) monitoring to ensure success; and (5) sharing the results.

## CONCLUSIONS

Implementing standardized postoperative inpatient clinical practice guidelines for patients following the Fontan procedure reduced practice variation and decreased hospital LOS. In addition, quality of care is maintained as evidenced by similar 30-day readmissions pre- and postguidelines.

## DISCLOSURE

The authors have no financial interest to declare in relation to the content of this article.
